# Sequence variations in the ETEC CS6 operon affect transcript and protein expression

**DOI:** 10.1080/21505594.2021.1981000

**Published:** 2021-10-21

**Authors:** Jonathan Moon, Eileen M. Barry

**Affiliations:** Center for Vaccine Development, University of Maryland School of Medicine, Baltimore, MD, USA

**Keywords:** ETEC, CS6, expression, regulation, vaccine

## Abstract

Enterotoxigenic *Escherichia coli* (ETEC) is a leading cause of diarrheal disease in developing nations where it accounts for a significant disease burden in children between the ages of 0 to 59 months. It is also the number one bacterial causative agent of traveler’s diarrhea. ETEC infects hosts through the fecal-oral route and utilizes colonization factors (CF) to adhere within the small intestine. Over 25 CFs have been identified; 7 are considered major CFs and a vaccine targeting these is predicted to provide protection against up to 66% of ETEC associated disease. Coli Surface Antigen 6 (CS6) is a major CF and is associated with disease-causing ETEC isolates. Analysis of the CS6 operon sequence led to the identification of two regions of variability among clinical isolates which we predicted exert effects on CS6 transcript and protein expression. A total of 7 recombinant *E. coli* strains were engineered to encode the CS6 operon in wild-type, hybrid, and mutant configurations. Western blot analysis and RT-qPCR provided evidence to support the importance of an intergenic hairpin structure on CS6 expression. Our results reveal the significance of CS6 sequence selection regarding ETEC vaccine development and present novel information regarding CS6 sequence variation in WT ETEC strains.

## Introduction

Enterotoxigenic *Escherichia coli* (ETEC) is a noninvasive enteric pathogen that is a significant cause of moderate-to-severe diarrheal disease (MSD) in children under five years of age in low resource regions. It is also a significant cause of traveler’s diarrhea [1–4]. ETEC infects hosts through the fecal-oral route and colonizes the small intestine by utilizing colonization factors (CF) to adhere to intestinal epithelial cells. Adherence within the gut is required for successful ETEC infection and it has been shown that CF-specific antibodies can inhibit colonization and prevent disease [5,6]. Subsequent delivery of heat-labile (LT) and/or heat-stable (ST) toxin increases the production of second messenger cyclic nucleotides within intestinal epithelial cells resulting in ion efflux [7–9]. This shift in the osmotic gradient results in excretion of water into the intestinal lumen and leads to the common clinical presentation of watery diarrhea [10]. Over 25 CFs have been identified; seven are classified as major CFs including CFA/I, and CS1-CS6; it has been estimated that a vaccine targeting the major CFs could provide protection against up to 66% of ETEC-associated disease [11]. Although multiple ETEC vaccine development strategies are ongoing, no FDA-approved ETEC vaccine currently exists [11–14].

Coli Surface Antigen 6 (CS6) is categorized as a major CF and multiple ETEC vaccine strategies have incorporated or targeted CS6 through a variety of approaches [15–17]. The CS6 operon is composed of four genes: *cssA* and *cssB* encode two structural subunits of 15 kDa and 16 kDa, respectively. *cssC* and *cssD* encode a chaperone and usher protein, respectively [18]. The crystal structure of both subunits was resolved elucidating that the two subunits which comprise the CS6 antigen are present in a 1:1 stoichiometric ratio linked by donor strand complementation [19]. Previous characterization of the CS6 antigen implicated fibronectin and sulfatide as host receptors within the gut [19,20]. Evidence supports the presence of multiple alleles of *cssA* and *cssB* in wild-type (WT) isolates and that SNPs in the sequences of these genes impact binding efficiency to Caco-2 cells [21]. Little information is available regarding other variations within the sequence of the CS6 operon and the resulting effects on CS6 expression although it has been suggested that CS6 gene expression may be regulated by a negative feedback loop [22].

Our analysis of the CS6 operon led to the identification of two regions which display heterogeneity in WT isolates. The first region occurs between the genes *cssB* and *cssC* where a stem-loop structure is predicted to form. The second variation is in the length of the *cssD* gene which may be truncated at the 3ʹ terminus resulting in the loss of another stem-loop structure. Stem-loop structures form as a result of intramolecular base pairing that result in secondary structures that have been shown to play a role in regulation [23]. Stem-loops acting as regulators of CF expression through enhancement of mRNA stability has previously been described [24], however secondary structures encoded within CF operons are largely uncharacterized. Based on preliminary CS6 expression data in ETEC isolates, we hypothesized that these regions of variability may contribute to differential CS6 expression. Recombinant plasmids encoding WT, hybrid, and mutant CS6 operons were engineered and confirmed the importance of these regions on CS6 expression. Ultimately, we endeavor to identify factors that affect CS6 expression and their contribution to optimization of CS6 expression in the context of a live-attenuated multivalent vaccine [25].

## Materials and methods

### Bacterial strains and culture conditions

Table 1 lists the strains and plasmids used in this study. Recombinant CS6 operons were cloned in DH5α and grown at 30°C on LB agar or in LB broth culture in the presence of 100 µg/mL carbenicillin or kanamycin. ETEC strains were grown on CFA agar (2% agar) or in CFA broth (1% Casamino acids, 0.15% Yeast Extract, 0.005% MgSO_4_, 0.0005% MnCl_2_) at 37°C [26,27].

**Table 1.** Strains used in this study

Strain Name	Strain Derivation	Description
CS6WT1	DH5α(pGRG-mLpp-CS6.1)	Small intergenic hairpin; Short cssD
CS6WT2	DH5α(pGRG-mLpp-CS6.2)	Large intergenic hairpin; Long cssD
CS6H1	DH5α(pGRG-mLpp-CS6.3)	Small intergenic hairpin; Long cssD
CS6H2	DH5α(pGRG-mLpp-CS6.4)	Large intergenic hairpin; Short cssD
CS6M1	DH5α(pGRG-mLpp-CS6.5)	Altered palindromic sequence of intergenic hairpin; no stem-loop formation
CS6M2	DH5α(pGRG-mLpp-CS6.6)	No intergenic region between *cssB* and *cssC*
CS6M3	DH5α(pGRG-mLpp-CS6.7)	PCR mutation in palindromic sequence of stem-loop; deletion of two bases
-	DH5α(pZA112-mLpp-CS6)	CS6 vector cloned from ETEC strain E17018A
ETEC 214–4	-	Wild-type ETEC (Group 1 CS6)
ETEC E17018A	-	Wild-type ETEC (Group 2 CS6)
-	DH5α(pGRG-mLpp)	Vector; CS6 Negative Control

### Sequencing of ETEC isolates

The primers CS601F and CS601R were used with the Roche Expand Long Template PCR kit (RocheLife Sciences, Mannheim, Germany) to amplify a 2195bp PCR product containing both the *cssB* and *cssC* genes. The primer CS6JF1 was used with either the primer cssD_long_R1 or cssD_short_R1 to amplify a 340bp or 328bp product, respectively, from 23 CS6 encoding ETEC isolates (Supplementary Figure 1). These PCR products were then sequenced to enable categorization of strains. Ninteen published CS6 sequences were also assessed and categorized [28].

### Molecular genetic techniques and construction of strains

The primers used to amplify the CS6 operon were generated by Integrated DNA Technologies (Coralville, IA) and are listed in Table 2. PCR reagents were purchased from Roche Life Sciences (Mannheim, Germany) and restriction enzymes were purchased from New England Biolabs (Ipswich, MA).

**Table 2.** Primers used in this study

Primer	Description	Sequence (5ʹ – 3ʹ)
cssA-F	*cssA* forward primer	TAACTCGAGATGAAGAAAACAATTGGTTTAATTCTAATTCTTG
cssB-R	*cssB* reverse primer	TAAGGCGCGCCTTAATTGCTGTAAAATGATACAGTCAAATTTCCTG
cssC-F1	*cssC* forward – no intergenic region	TAAGGCGCGCCATGAAATCAAAGTTAATTATACTATTGACGTTAGTGCCATTTTC
cssC-F2	*cssC* forward – small stem- loop	TAAGGCGCGCCAAAAAGGCCGCATTATTGCGGCCATTGACGATACTGCTAGGCAAAAAT
cssC-F3	*cssC* forward – altered stem- loop	TAAGGCGCGCCAAAAAGTCATGATTATTGATTGCGGCCATTGACGATACTGCCAG
cssC-F4	*cssC* forward – large stem- loop	TAAGGCGCGCCAAAAAGGCCGCATTATTGATTGCGGCCATTGACGATACTGCCAGGCAAA
cssD-R1	*cssD* reverse – short *cssD*	GGCGCGCCCTAACATTGTTTATTTACAACAGATAATTGTTTGCTAG
cssD-R2	*cssD* reverse – long *cssD*	TTAGGCGCGCCTTAGTCTCCAGAATTTTCGGGGCG
cssA -F-qPCR	*cssA* forward RT-qPCR	GGACGACTCGTAAATACCGCT
cssA-R-qPCR	*cssA* reverse RT-qPCR	TTAGGCGTAACCTCTGCACC
cssB-F-qPCR	*cssB* forward RT-qPCR	TCTGGACAGCAGATCGGAAAG
cssB-R-qPCR	*cssB* reverse RT-qPCR	TGCCCTGCCATAAACTTACCA
cssC-F-qPCR	*cssC* forward RT-qPCR	TGAATCAATGCCACCAACAGA
cssC-R-qPCR	*cssC* reverse RT-qPCR	AATGCATCCCCGAATGCTGA
cssD-F-qPCR	*cssD* forward RT-qPCR	CGGCAACCAGTTCTGTAGGT
cssD-R-qPCR	*cssD* reverse RT-qPCR	TACGGGTCGTTCTGTTCTGC
rpoD-F	*rpoD* forward RT-qPCR	GAGCAAGGCTATCTGACCTATG
rpoD-R	*rpoD* reverse RT-qPCR	GCCCATGTCGTTGATTTG
CS601F	*cssB-cssC* forward primer	TGGTGCAGAGGTTACACCTAATC
CS601R	*cssB-cssC* reverse primer	GAGAGTCTGAATCAGCCACTCCATG
cssD_long_R1	Longer cssD reverse primer	CTCTTTCTCAGGAAGTTTAGTCTCCAGAATTTTCGG
cssD_short_R1	Shorter cssD reverse primer	CACATGTTCTACTAATTGGATGCACTACCTAAC

The CS6 operon was amplified by PCR as a 4294bp fragment from the ETEC strain E17018A. The amplified product was ligated into the TOPO-XL cloning vector. The CS6 insert was then excised with XhoI/SpeI and ligated into pZA112 (formerly named pGA2) [29] to create the plasmid pZA112-mLpp-CS6. The operon was cloned under the control of a mutated Lpp promoter (PmLpp) to enhance transcription [30]. To develop the recombinant CS6 operons, the genes *cssA* and *cssB* were amplified from pZA112-mLpp-CS6 as a single 986bp fragment using the Roche Expand Long Template PCR kit (Roche Life Sciences, Mannheim, Germany) with XhoI and AscI ends. This PCR product was digested using XhoI and AscI; T4 DNA Ligase (New England Biolabs, Ipswich, MA)) was used to ligate the fragment into the vector pGRG-mLpp to yield pGRG-mLpp-*cssA-cssB* [31]. The *cssC* and *cssD* genes, including the non-coding region between *cssB* and *cssC*, were subsequently amplified from pZA112-mLpp-CS6 as either a 3,139bp or 3,167bp product with flanking AscI ends. The 3,139bp product encodes the smaller stem-loop structure and the shorter *cssD* gene and was amplified using the primers cssC-F2 and cssD-R1 to create the construct CS6WT1. The construct CS6WT2 was created using the 3,167bp product, which encodes the longer versions of each region, and was amplified with the primers cssC-F4 and cssD-R2. Two hybrid CS6 constructs were also engineered one of which, CS6H1, contained the smaller stem-loop and longer *cssD* (3,163bp), amplified with primers cssC-F2 and cssD-R2, and the other, CS6H2, containing the larger stem-loop and shorter *cssD* (3,143bp) was amplified with primers cssC-F4 and cssD-R1. Alternative CS6 operon constructs were created in which the stem-loop between *cssB* and *cssC* was mutated to not form or was deleted entirely. The alternative construct, CS6M1, was engineered by changing the nucleotides in one half of the stem-loop sequence such that the structure would no longer form while leaving the entire intergenic region intact. This was accomplished during PCR amplification of the *cssC* and *cssD* genes using the forward primer cssC-F3, in which one half of the palindromic stem sequence was altered from GGCCGC to GTCATG, and the reverse primer cssD-R1. In the alternative construct CS6M2, the entire intergenic region was deleted by amplifying the *cssC* and *cssD* genes with the forward primer cssC-F1 which binds at the *cssC* start codon to exclude the intergenic sequence completely and the reverse primer cssD-R1. Finally, the construct CS6M3 acquired a mutation (G and C deletion from one of two palindromic sequences) within the stem-loop during PCR with the primers cssC-F2 and cssD-R1 that increased the predicted Gibbs Free Energy (*ΔG*) of the structure thus reducing the likeliness of its random formation. All *cssC-cssD* PCR products were digested and ligated into the vector pGRG-mLpp-*cssA-cssB*. Correct cloning was confirmed by colony PCR using the primers CS601F and CS601R and positive transformants were isolated and stored in LB stocks with 20% glycerol at −80°C. Plasmids were extracted using the GeneJet Plasmid Miniprep Kit (Thermo Fisher Scientific, Waltham, MA), and sequenced for confirmation.

### RT-qPCR

Each recombinant CS6 strain was grown from frozen stocks in LB broth culture (Becton Dickinson, Franklin Lakes, NJ)). Subcultures were grown to an OD_600_ of 0.5. 10 mL of each culture was sampled for total RNA isolation. Bacterial pellets were resuspended in TriPure Isolation Reagent (Roche Life Sciences, Mannheim, Germany), extracted with chloroform and precipitated with isopropanol. Each RNA pellet was resuspended in nuclease-free water (Thermo Fisher Scientific, Waltham, MA) plus 1 µL of RNase Out (Thermo Fisher Scientific, Waltham, MA)). Contaminating DNA was removed from the total RNA sample using the Turbo DNA-*free* kit (Thermo Fisher Scientific, Waltham, MA) and the absence of contaminating DNA was confirmed by endpoint PCR. cDNA was generated using the qScript cDNA Synthesis Kit (Quantabio, Beverly, MA)

qPCR primer sequences (Table 2) were generated using the NCBI primer design tool. Each gene was analyzed in triplicate from two biological replicate samples using FastStart Universal SYBR Green Master Mix (Rox) (Roche Life Sciences, Mannheim, Germany)). The gene *rpoD* was used to normalize across samples. The fold change of each transcript was calculated using a ΔΔCt analysis: the Ct value recorded for each CS6 sample was subtracted from the average Ct value of the *rpoD* replicates to give the normalized ΔCt value. CS6WT2 was used as the control sample and the triplicate ΔCt values from each individual CS6 gene detected from the Group 2 construct were averaged to give an average control value. Then, the average control value was subtracted from the ΔCt value of each replicate sample from each other construct variation to give the ΔΔCt value for each replicate sample. The fold change in each sample was then calculated as 2^−ΔΔCt^ and the average fold change from triplicate wells was then calculated from these values which allowed for comparison of relative transcript quantity.

A primer efficiency curve was also carried out using a 1:10 serial dilution of CS6WT1 cDNA to a final dilution of 1:10,000,000. Reactions were prepared as previously stated. The average Ct of the replicate wells analyzing each CS6 gene were then plotted linearly against the respective dilution factor.

### Western blot analysis

Whole cell bacterial lysates were prepared for western blot analysis as follows. Bacteria were harvested from overnight growth on agar plates into 1xDPBS (Corning, Corning, NY) and the OD_600_ was used to normalize to an OD of 3.0. The normalized suspensions were then mixed 1:1 with 2x Laemmli Sample Buffer (0.125 M Tris pH 6.8, 4% SDS, 20% Glycerol, 0.2% Bromophenol Blue, 10% β-mercaptoethanol). Proteins were separated on a 12% Mini-Protean TGX Precast Gel (Bio-Rad, Hercules, CA) before being transferred to a PVDF membrane. The membrane was blocked in a 10%(w/v) nonfat milk buffer in 1xDPBS and then incubated with polyclonal Rabbit α-CS6 sera (Rockland Immunochemicals, Limerick, PA) in 10% (w/v) nonfat milk buffer overnight at 4°C on an orbital shaker. Following washing, the membrane was incubated with secondary antibody Goat α-Rabbit 680 nm (Invitrogen, Carlsbad, CA) and proteins visualized using the 700 nm wavelength setting on an LI-COR Odyssey Laser Scanner.

## Results

### Sequence analysis of ETEC and recombinant strains encoding CS6

**Figure 1.**
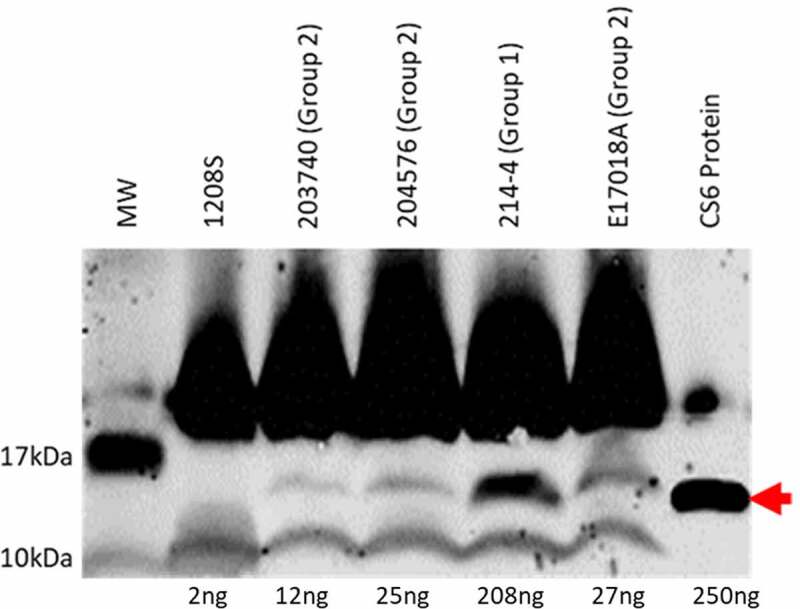
Western blot analysis of four different CS6^+^ ETEC strains. Expression of CS6 was determined by western blot analysis of whole cell lysates. Strains 203740, 204576, and E17018A were categorized as Group 2 ETEC strains and share the same CS6 operon structure. 214–4 was categorized as Group 1 and is distinct in sequence from Group 2 strains. Protein (CS6) amounts were quantified by densitometric analysis based on the single CS6 purified protein standard (lane 7) and the negative control 1208S (lane 2) which is an attenuated *Shigella flexneri* strain

**Figure 2.**
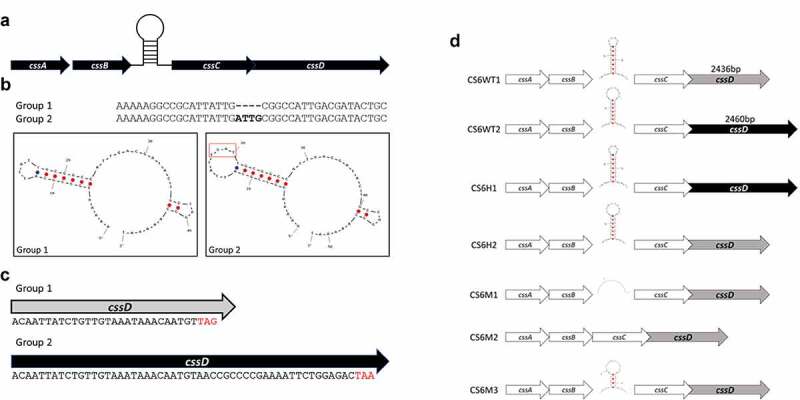
CS6 operon structures. a) Organization of the CS6 operon demonstrating the stem-loop structure the intergenic region between *cssB* and *cssC*. b) The sequence of the stem-loop structure denoting 4-bp smaller loop structure in Group 1 CS6 compared to Group 2 CS6. c) In Group 1 CS6, the final gene in the operon, *cssD*, is truncated by 24bp compared to Group 2 CS6 and exhibits no identifiable downstream sequence homology. d) Schematic display of the seven recombinant CS6 operons. CS6WT1 and CS6WT2 are found in WT-ETEC strains. CS6H1 and CS6H2 were engineered as hybrids and were not observed in any WT strains. In CS6M1, one half of the palindromic sequence was mutated such that the hairpin structure does not form. CS6M2 does not contain any intergenic region between *cssB* and *cssC*. CS6M3 acquired two deletions in the palindromic sequence during PCR amplification and forms a shorter stem comprised of 4 bases rather than the typical 6–7

Preliminary characterization of a subset of CS6 encoding ETEC isolates demonstrated differential CS6 expression under identical growth conditions; ETEC strain 214–4 expressed a higher level of CS6 than strains E17018A, 203740 and 204576 (Figure 1). Sequence analysis of ETEC isolates 214–4 and E17018A revealed two regions of variation within the CS6 operon (Figure 2). The first region occurs in a noncoding sequence between the genes *cssB* and *cssC*. In 214–4, this noncoding sequence is comprised of 48bp while in E17018A the sequence totals 52bp. Importantly, the difference of 4bp corresponds to the formation of a stem-loop structure containing either 4 or 8 bases within the loop (Figure 2(b)). This structural variation of the stem- loop may impact its formation and stability as indicated by the Gibbs Free Energy (ΔG) which was calculated to be −10.3 and −9.5 in WT1 strain 214–4 and WT2 strain E17018A, respectively.

The second variable region occurs at the 3ʹ terminus of the *cssD* gene. In 214–4, the gene totals 2436bp while in E17018A it measures 2460bp (Figure 2(c)). The difference of 24bp translates to an additional 8 amino acids at the carboxyl terminus of CssD in E17018A. Also, within this extra 24bp sequence another stem-loop structure is predicted to form in E17018A that is absent in the 214–4 sequence.

We analyzed the CS6 sequences of a total of 42 unique ETEC isolates from 8 geographically distinct locations to assess the distribution of the motifs we observed in 214–4 and E17018A (Table 3). Of the 42 strains analyzed, 9 encoded the CS6 operon with the smaller stem-loop and shorter *cssD*, consistent with ETEC strain 214–4, and were classified as Group 1 strains. The remaining 33 strains were classified as Group 2 strains and encoded the CS6 operon containing the larger stem-loop and longer *cssD* as observed in ETEC strains E17018A, 203740, and 204576. All strains examined in this subset conformed to either the Group 1 or Group 2 classification. Further evaluation revealed that strains encoding the STh and LT toxins were present in both groups, but strains encoding STp were limited to Group 1. Strains in both groups encoded operons for multiple CFs; we noted that no Group 1 strains contained genes that encode CS5 and that no Group 2 strains encoded CS4. These trends were consistent within the subset of strains analyzed.

**Table 3.** Distribution of CS6 operon motifs in geographically diverse CS6 ETEC isolates

CS6 Operon Group	Location	Colonization Factors	Toxin Profile
**Group 1**			
201446	Mali	CS4, CS6	STp, LT
302054	Mozambique	CS6, CS21	STp, LT
401061	Kenya	CS4, CS6, CS21	STp, LT
503046	India	CS4, CS6, CS21	STh
503458	India	CS4, CS6, CS21	STh
510016	India	CS4, CS6, CS21	STh
520873	India	CS4, CS6, CS21	STh
214–4	Mexico	CS6, CS21	STp
E8775	Mexico	CS4, CS6	ST, LT
**Group 2**			
100137	Gambia	CS5, CS6	LT
100345	Gambia	CS5, CS6	STh
100491	Gambia	CS6	STh, LT
103605	Gambia	CS5, CS6	STh
120899	Gambia	CS5, CS6	STh
200006	Mali	CS6, CS21	LT
200065	Mali	CS5, CS6	STh
203740	Mali	CS6	STh
204446	Mali	CS5, CS6	STh, LT
204576	Mali	CS5, CS6	STh
300006	Mozambique	CS5, CS6	STh, LT
300007	Mozambique	CS5, CS6	STh, LT
300239	Mozambique	CS6, CS14, CS21	LT
400588	Kenya	CS5, CS6, CS14	STh, LT
400650	Kenya	CS6	STh
401068	Kenya	CS6, CS14, CS21	LT
500465	India	CS6, CS21	STh
500819	India	CS5, CS6	LT
503025	India	CS6, CS21	STh
503440	India	CS6, CS21	STh
503663	India	CS6	STh, LT
503829	India	CS6, CS21	STh
504211	India	CS6, CS21	STh
504237	India	CS5, CS6	STh
600489	Bangladesh	CS5, CS6	STh, LT
602354	Bangladesh	CS5, CS6	STh
604113	Bangladesh	CS6, CS21	LT
E10703	Bangladesh	CS5, CS6	ST
E17018A	Bangladesh	CS5, CS6	ST
700006	Pakistan	CS5, CS6	STh, LT
700173	Pakistan	CS5, CS6, CS14	STh, LT
700360	Pakistan	CS6	LT
703322	Pakistan	CS5, CS6	LT

Seven recombinant constructs were engineered in an identical plasmid-based system to assess the potential effects that these sequence variations confer on CS6 expression (Figure 2(d)). The operons were cloned behind a constitutive promoter to remove any impact of upstream regulation. CS6WT1 and CS6WT2 correspond to the Group 1 and Group 2 WT operons, respectively. CS6H1 and CS6H2 are hybrid operons which contain the two combinations of Group 1 and Group 2 stem-loop and *cssD* motifs. CS6M1, CS6M2, and CS6M3 all include the Group 1 *cssD* motif (2436bp) but are mutated in the stem-loop sequence. In CS6M1 the nucleotides in one half of the *cssB-cssC* stem-loop were altered such that the structure would no longer form. In CS6M2, the entire noncoding region between *cssB* and *cssC* was deleted. CS6M3 contains two deletion mutations in the stem-loop’s palindromic sequence which results in formation of a 4bp stem as opposed to 7bp in the WT and hybrid configurations and an intergenic sequence composed of 46bp.

### CS6 protein expression

Expression of the CS6 antigen in WT and recombinant strains was assessed by western blot analysis. Bands were quantitatively assessed using densitometric analysis based on a single CS6 protein standard. Initial analysis of WT ETEC strains demonstrated a 7.7-fold increase in CS6 expression in the Group 1 strain 214–4 compared to the Group 2 strain E17018A (Figure 1).

**Figure 3.**
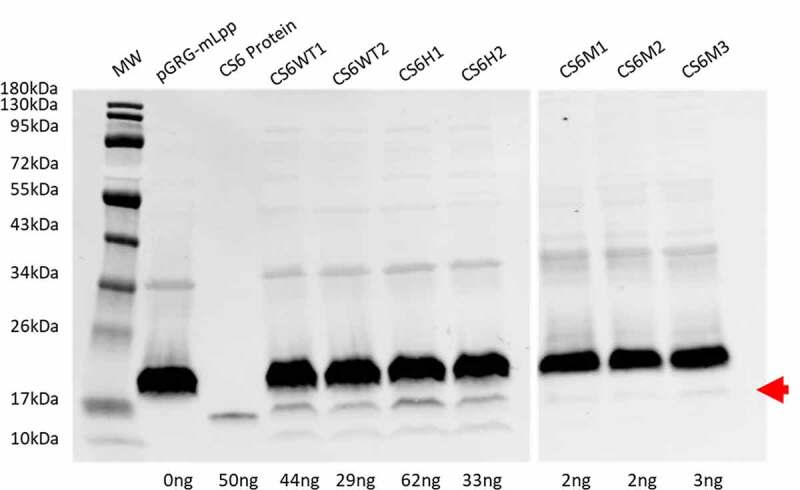
CS6 expression. Expression of CS6 from the recombinant operons expressed in DH5α was determined by western blot analysis of whole cell lysates. Proteins (CS6) were quantified by densitometry based on the single CS6 protein standard (lane 3) and the negative control pGRG-mLpp. CS6M1, CS6M2, and CS6M3 were run on a separate blot with a single CS6 protein standard used for densitometric analysis

Expression of CS6 was compared among the recombinant CS6 constructs (Figure 3). CS6H1, which encodes the Group 1 stem-loop and Group 2 *cssD*, exhibited the highest levels of CS6 expression; expression was 40% greater than CS6WT1 and 110% greater than CS6WT2. CS6H2 which encodes the Group 2 stem-loop and Group 1 *cssD* expressed ~50% of the level of CS6 in CS6H1 and was intermediate between CS6WT1 and CS6WT2. CS6 expression in all three mutant constructs, CS6M1, CS6M2, and CS6M3 was very low and was approximately comparable between all three mutants.

### CS6 transcript expression

**Figure 4.**
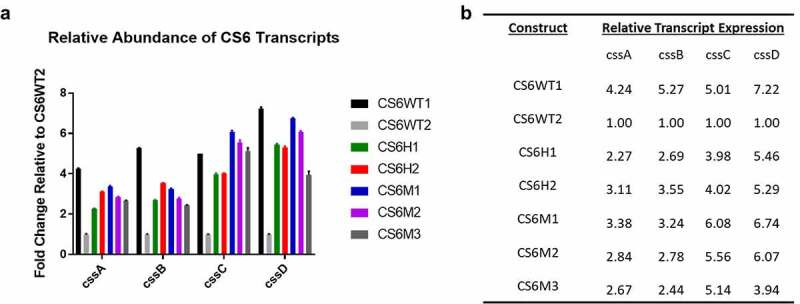
A) RT-qPCR analysis of CS6 transcript abundance compared to CS6WT2. Strains were grown to OD_600_ 0.5 in LB broth and harvested to isolate total RNA. b) Relative fold change values of CS6 transcripts in each recombinant CS6 strain

The observed range of CS6 antigen expression in the recombinant CS6 constructs prompted investigation into CS6 mRNA expression as stem-loop structures have been implicated in the regulation of both transcription and translation [24,32]. We quantified transcripts of all four CS6 genes using RT-qPCR and calculated the relative amount of transcript expressed through a ΔΔCt analysis. Ct values determined for each construct were generated relative to those values calculated for CS6WT2 (Figure 4). Overall, expression of all four *css* genes was higher in all engineered constructs relative to CS6WT2. These data showed that, on average, CS6WT1 expressed 4.24-fold more *cssA* transcript, 5.27-fold more *cssB* transcript, 5.01-fold more *cssC* transcript, and 7.22-fold more *cssD* transcript than CS6WT2. CS6H1 and CS6H2 expressed 2.27 and 3.11-fold more *cssA* transcript, 2.69 and 3.55-fold more *cssB* transcript, 3.98 and 4.02-fold more *cssC* transcript, and 5.46 and 5.29-fold more *cssD* transcript, respectively. The mutants that abolished the *cssB-cssC* intergenic stem-loop structure (CS6M1 and CS6M2) or reduced the stem length (CS6M3) had higher levels of all transcripts than CS6WT2. CS6M1, CS6M2, and CS6M3 also expressed 3.38, 2.84, and 2.67-fold greater amounts of *cssA*, respectively. *cssB* transcripts in the mutant constructs were elevated by 3.24, 2.78, and 2.44-fold, respectively. cssC transcripts were increased by 6.08, 5.56, and 5.14-fold, respectively. Finally, cssD transcripts were greater by 6.74, 6.07, and 3.94-fold, respectively when compared to CS6WT2 transcript quantities (Figure 4(a)).

Primer efficiency was calculated to be 102.46%, 99.15%, 91.97%, 100.37%, and 92.10% for the genes *cssA, cssB, cssC, cssD* and *rpoD*, respectively (Supplementary Figure 2).

## Discussion

The regulation of ETEC CF expression is affected by environmental ques via upstream sequences and regulators including *rns* [33–35]. Additionally, regulation at the transcriptional level is impacted by intergenic sequences including those that form stem-loop structures [24,32,35]. Stem-loops have also been described in the sequences of the genes *eltA* and *eltB* which encode subunits of the LT toxin and are suggested to have a regulatory role in expression providing some precedence for stem-loop structures as regulators in ETEC [36]. Understanding this regulation lends insight into the role of CFs during disease and provides information for optimizing their expression in vaccine constructs. The Center for Vaccine Development and Global Health has an ongoing program to develop a multivalent *Shigella*-ETEC vaccine that is composed of live attenuated *Shigella* strains expressing immunogenic ETEC antigens including major CFs [37,38]. This strategy requires optimization of heterologous antigen expression. CS6 is a major CF and is a critical component of a broadly protective vaccine against ETEC [11].

We initially observed variable CS6 expression in WT ETEC strains which led us to examine the sequences of the CS6 operons. Several SNPs were present in coding sequences, but interestingly we identified two regions within the operon that exhibited more pronounced divergence. Sequence analysis of a selection of clinical ETEC isolates [28] revealed that isolates could be categorized based on their CS6 operon profile as defined by the variable regions. We found that the smaller stem-loop was always present in CS6 operons that also had the shorter *cssD* gene (Group 1) and that the larger stem-loop was always present with the longer *cssD* gene (Group 2). This association occurred regardless of the geographical location from which the strain was isolated or the strains’ toxin profile. We noted that the total CF profile of the strains did not associate with the CS6 operon variation (Group 1 or Group 2) although none of the Group 1 strains we analyzed encoded CS5 and none of the Group 2 strains encoded CS4. These data suggest that at least two variations of the CS6 operon exist in WT strains and are not geographically or genotypically restricted.

Western blot analysis of recombinant CS6 strains provided evidence to support a contribution of the stem- loop, which forms between the genes *cssB* and *cssC*, to regulation of expression. CS6WT1 and CS6H1 strains encode the smaller version of this structure, which contains four bases within the loop. These strains expressed more CS6 protein than constructs encoding the larger stem-loop containing eight bases in the loop (CS6WT2 and CS6H2). When the stem-loop sequence was altered or deleted, we observed a dramatic decrease in CS6 protein expression. These results suggest that there is an optimal configuration of the stem- loop which intrinsically promotes greater CS6 protein expression. This could be due to differences in how the mRNA folds and fits into the ribosome; further characterization of the mRNA’s structure is necessary to evaluate this.

Although we detected differential protein expression across the recombinant strains, RT-qPCR analysis provided evidence that all CS6 transcripts were elevated to similar levels in recombinant CS6 strains when compared to the Group 2 recombinant strain, CS6WT2. We were surprised to find that CS6M1, CS6M2, and CS6M3 also expressed more CS6 transcripts. We hypothesize that when this structure is absent entirely there is a lack of inhibitory regulation of CS6 transcription, potentially due to the absence of physical interference by the secondary structure. The increase in *css* transcription by the mutant operons in conjunction with negligible protein expression, supports the requirement for the stem-loop structure for RNA stability and/or protein translation. The decoupling of transcript quantity and protein expression suggests another potential regulatory role of the two variable regions that we identified in the CS6 operon. Multiple post-transcriptional effects have been attributed to stem-loop structures including regulation of gene expression through transcript stability [24,35] and it has been shown that mutations resulting in the loss of specific stem-loops can impact protein expression [32]. Furthermore, secondary structures such as stem-loops and cruciforms are known to form in DNA and can exert a variety of effects, including sterically impeding binding of RNA polymerases or other transcription factors resulting in reduced transcriptional activity [39]. .

The effect of the truncated *cssD* gene is not entirely clear. However, the increase in transcription in CS6H2 which encodes the same *cssB-cssC* stem-loop as CS6WT2, but encodes the shorter *cssD*, supports its role in regulation. We posit that the lack of the 3ʹ terminal stem-loop alters the folding of the molecule such that negative regulation of transcription is reduced.

Our data suggest a role in regulation of CS6 expression by two secondary structures we identified in this study potentially due to alterations in the conformation of the CS6 mRNA.

CS6 is an important antigen in the development of an ETEC vaccine as it is one of the most common CFs associated with ETEC disease. There have been various ETEC vaccine candidates developed which incorporate CS6 [15,16,40], however no FDA licensed vaccine currently exists. We showed that the sequence of the stem-loop and of the *cssD* gene impact both CS6 transcript and protein expression in recombinant *E. coli* strains which share identical genetic backgrounds. Our results provide support for the implementation of one version of the CS6 operon for use in vaccine design for optimization of high level CS6 expression. Further characterization of the CS6 operon mRNA and visualization of recombinant strains by immunogold electron microscopy may yield more insight into differences in antigen export and surface expression which may provide further support for identification of a superior CS6 operon allele for implementation in ETEC vaccine design.

## Supplementary Material

Supplemental Material

## Data Availability

Supporting sequence data of ETEC strains utilized in this study are available online through GenBank at https://www.ncbi.nlm.nih.gov/nuccore/?linkname=pubmed_nuccore&from_uid=30944874 [28].
